# Tracking and predicting U.S. influenza activity with a real-time surveillance network

**DOI:** 10.1371/journal.pcbi.1008180

**Published:** 2020-11-02

**Authors:** Sequoia I. Leuba, Reza Yaesoubi, Marina Antillon, Ted Cohen, Christoph Zimmer

**Affiliations:** 1 Epidemiology of Microbial Diseases, Yale School of Public Health, New Haven, CT, USA; 2 Health Policy and Management, Yale School of Public Health, New Haven, CT, USA; 3 Household Economics and Health Systems Research Unit, Swiss Tropical and Public Health Institute, Basel, Switzerland; 4 University of Basel, Basel, Switzerland; National Institutes of Health, UNITED STATES

## Abstract

Each year in the United States, influenza causes illness in 9.2 to 35.6 million individuals and is responsible for 12,000 to 56,000 deaths. The U.S. Centers for Disease Control and Prevention (CDC) tracks influenza activity through a national surveillance network. These data are only available after a delay of 1 to 2 weeks, and thus influenza epidemiologists and transmission modelers have explored the use of other data sources to produce more timely estimates and predictions of influenza activity. We evaluated whether data collected from a national commercial network of influenza diagnostic machines could produce valid estimates of the current burden and help to predict influenza trends in the United States. Quidel Corporation provided us with de-identified influenza test results transmitted in real-time from a national network of influenza test machines called the Influenza Test System (ITS). We used this ITS dataset to estimate and predict influenza-like illness (ILI) activity in the United States over the 2015-2016 and 2016-2017 influenza seasons. First, we developed linear logistic models on national and regional geographic scales that accurately estimated two CDC influenza metrics: the proportion of influenza test results that are positive and the proportion of physician visits that are ILI-related. We then used our estimated ILI-related proportion of physician visits in transmission models to produce improved predictions of influenza trends in the United States at both the regional and national scale. These findings suggest that ITS can be leveraged to improve “nowcasts” and short-term forecasts of U.S. influenza activity.

## Author summary

The United States Centers for Disease Control and Prevention (CDC) tracks influenza activity through a national surveillance system. However, the CDC influenza surveillance data are subject to a 1 to 2 week reporting delay, which limits how such information can be used to assess the current burden of disease and to make timely projections of the trajectory of the epidemic. Researchers have previously used indirect signals of influenza activity such as Google search queries for influenza-related terms or Twitter posts to develop more timely estimates of influenza burden and improved model-based forecasts. However, these indirect signals are subject to behavioral changes not related to influenza activity, and thus may provide inaccurate estimates and projections. We used a new real-time data source with high geographic resolution that is directly related to influenza activity: influenza test results provided through a national network of commercial influenza diagnostic test machines. We used these influenza test results to accurately estimate the current burden of influenza and improve real-time model projections of influenza burden in the United States.

## Introduction

The U.S. Centers for Disease Control and Prevention (CDC) estimates that influenza is responsible annually for 9.2 to 35.6 million illnesses and 12,000 to 56,000 deaths in the United States [1]. Accurate and timely estimates of influenza activity are essential for public health planning and evaluation of vaccine effectiveness.

The CDC tracks influenza activity through several sources of data which contribute to a national surveillance network consisting of the World Health Organization Collaborating Laboratories System and the National Respiratory and Enteric Virus Surveillance System (WHO/NREVSS) [2]. These systems collect weekly records of the numbers and results of diagnostic tests for influenza from approximately 100 public health laboratories and 300 additional clinical laboratories [2]. Furthermore, U.S. Outpatient Influenza-like Illness Surveillance Network (ILINet) compiles weekly reports from over 2,800 healthcare providers on the total number of physician visits and number of these visits associated with an influenza-like illness (ILI), defined as fever and cough and/or sore throat without an identified cause other than influenza [2]. Additional data on influenza-related hospitalizations and deaths are collected through other systems [1]. In summary, this surveillance network provides weekly estimates of the number of influenza tests ordered, the number of these tests that confirm influenza infection, the number of physician visits, and the number of these physician visits that are related to an ILI [2].

The CDC data serve as the gold standard for national and sub-national influenza surveillance in the U.S. These data are subject to a 1 to 2 week reporting lag and are iteratively backfilled and revised [3]. Given this delay in reporting, there has been interest in understanding whether other data sources can provide more rapid estimates of current influenza activity (i.e., “nowcast”) and whether more timely information can be used to improve model-based forecasts [3–5]. Many recent investigations have focused on the use of internet activity (e.g., Google [4] and Wikipedia searches [5], and Twitter feeds [3]) as indirect signals of influenza. The validity of such indirect sources of evidence is questionable as they are subject to changing behaviors of the public that may not be caused by influenza activity [5–7].

In this study, we evaluated the utility of near real-time data collected from a national network of influenza diagnostic machines to estimate the current burden and make short- and medium-term forecasts of influenza activity. We hypothesize that these automated data on influenza test results can inform valid estimates of the current influenza burden and that the elimination of reporting delays will improve model-based forecasts.

Quidel Corporation manufactures diagnostic influenza test machines which are used nationally. These machines automatically transmit de-identified data on influenza tests to a dataset called the Influenza Test System (ITS), thus facilitating near real-time quantification of influenza test numbers and results at high spatial resolution [8].

In comparison to the surveillance data available through the CDC which provides information on sub-national levels and with a one-week reporting delay, the ITS dataset provides information on the zip-code level and in real-time, and thus has higher spatial resolution and reduced reporting delays. However, it is unknown whether this alternative data source of influenza activity can serve as a valid proxy for the official CDC data. Also, even if the ITS network can be used to validly estimate the CDC data, it is not clear whether higher spatial resolution and more rapid availability can improve model-based projections of influenza dynamics. In this study, we determined that ITS data can be used to “nowcast” the CDC ILI metrics currently used to track influenza activity. We then found that the earlier availability of such estimates lead to improvements in the performance of model-based projections of influenza epidemics.

## Methods

We received Influenza Test System (ITS) datasets and calculated the total number of flu tests, the number of positive flu tests, the total number of test machines, and the proportion of flu tests that are positive on national and sub-national levels. The ITS datasets include test location and result as well as patient age and gender. For our sub-national levels, we adopted the regions used by the CDC, i.e., regions as defined by the Health and Human Services (HHS) Regions [2] [S1 Text: Cleaning ITS]. We then compared the results from the ITS network to those from the CDC influenza surveillance system data by comparing the ITS model to a baseline model without ITS data using ANOVA.

The CDC publishes data on the numbers and results of influenza tests, collected from WHO/NREVSS, and on the number of physician visits, both related to and unrelated to ILI, compiled from ILINet [2]. WHO/NREVSS and ILINet datasets are reported by epidemiological week (epi week) [2], and thus, we conducted our analysis by epi week. We developed a volume metric to compare the number of tests recorded in the ITS network to the number of tests reported by the CDC influenza surveillance system. We determined sub-national and national volume of the ITS dataset by dividing the total number of ITS-reported influenza tests in the specified region per epi week by the total number of CDC-reported influenza tests in the same region per epi week, and then averaged this metric over the length of the analysis. While the volume metric only included information from the WHO/NREVSS system, we used it as a proxy for the entire CDC influenza surveillance system (WHO/NREVSS and ILINet) as ITS did not include corresponding information on physician visits as provided by ILINet. All statistical analysis was performed in R version 3.3.1 [9] and Mathematica version 10.4 [10].

### Nowcasting using ITS

We first evaluated whether ITS data could produce accurate real-time estimates of the CDC data on current influenza activity, and thus serve as a valid proxy for the gold-standard data. We estimated two CDC metrics: (Model 1) the proportion of diagnostic tests that are positive [11], and (Model 2) the weighted ILI-related proportion of physician visits [12–14]. The CDC weights the proportion of physician visits that are ILI-related, *ILI*_*prop*_, by state population [13].

We note that data recording for the ITS systems started at different time points for the HHS regions: while ITS provides data on most regions starting from epi week 36 in 2015, the data recording for Region 2 and Region 10 started later than the other regions. Region 2 data was used starting at epi week 13 in 2016, and Region 10 data was used starting from epi week 2 in 2016 [S1 Text: Cleaning ITS].

We used a linear logistic model to investigate the relationship between the proportion of diagnostic tests that are positive as recorded by ITS (*V*_*ppt*_) and the proportion of tests that are positive as reported by the CDC (*ILI*_*ppt*_) (Eq 1)

$$\begin{array}{c} \begin{array}{cc} logit(ILI_{ppt}\left(t_{a}\right)) & =\beta_{1}logit\left(V_{ppt}\left(t_{a}\right)\right)+\beta_{2}logit\left(ILI_{ppt}\left(t_{\left(a-1\right)}\right)\right)\\ & \;\;+\beta_{3}logit(|V_{ppt}\left(t_{\left(a-1\right)}\right)-ILI_{ppt}\left(t_{\left(a-1\right)}\right)\left|\right)\\ & \;\;+\epsilon \end{array} \end{array}$$ (1)

where *β*_1_, *β*_2_, and *β*_3_ are the coefficients; *t* is the time variable with the current epidemiological week as *t*_*a*_ and the previous epidemiological week as *t*_(*a*−1)_; and *ϵ* is the error term. The logit transform for the proportions facilitates the estimation of proportions using OLS linear regression. The previous week’s CDC estimates inform the intensity of positive tests of this particular season, while the current week’s Virena tests inform week-to-week changes. We corrected for the systematic difference between Virena and CDC using the difference observed in the previous week’s readings (*V*_*ppt*_(*t*_*a*−1_) and *ILI*_*ppt*_(*t*_*a*−1_)). We use the absolute difference between the previous week’s *V*_*ppt*_(*t*_*a*−1_) and *ILI*_*ppt*_(*t*_*a*−1_) because we were interested in the distance between the Virena and the CDC estimates. This also facilitates the use of the logit transformation which requires positive inputs.

We then estimated a measure of influenza-related care seeking, the weighted proportion of physician visits that are ILI-related. We used a linear logistic model to investigate the relationship between total number of influenza test results divided by the total number of test machines as recorded by ITS (*V*_*total*_) and the weighted proportion of all physician visits that are ILI-related as reported by the CDC (*ILI*_*prop*_) (Eq 2).

$$\begin{array}{c} \begin{array}{cc} logit(ILI_{prop}\left(t_{a}\right)) & =\beta_{1}\left(V_{total}\left(t_{a}\right)\right)+\beta_{2}logit\left(ILI_{prop}\left(t_{\left(a-1\right)}\right)\right)\\ & \;\;+\beta_{3}\left(V_{total}\left(t_{\left(a-1\right)}\right)\right)+\epsilon \end{array} \end{array}$$ (2)

where *β*_1_, *β*_2_, and *β*_3_ are the coefficients; *t* is the time variable with the current epidemiological week as *t*_*a*_ and the previous epidemiological week as *t*_(*a*−1)_; and *ϵ* is the error term. We developed the *V*_*total*_ metric as a way of standardizing the ITS data, since the number of test machines vary per region and the number of test results is dependent on the number of test machines.

### Forecasting using ITS

We investigated whether influenza burden estimates developed by real-time ITS data could improve the performance of epidemic model forecasting. We used a humidity-based susceptible-infectious-recovered-susceptible (SIRS) transmission dynamic model [15], the ITS estimate for *ILI*_*prop*_ as a proxy for the new weekly cases, and a previously established framework for calibration on prediction to develop forecasts of the weighted ILI-related proportion of physician visits [16, 17].

We modified the model described in Yang et al., 2014 [15] with the technical specifications described in Zimmer et al., 2018 [16] [S1 Text: Computational Model]. Like previously published models, we did not distinguish between different strains and non-influenza sources for ILI [5].

This previously established framework for calibration and prediction summarizes initial knowledge on the epidemic parameters in a prior distribution which is iteratively updated with the probability of each new observation [16, 17]. This probability is calculated with the help of a linear noise approximation to capture the fluctuations and a state updating mechanism to incorporate information from the most recent observations [18]. Forecasts are made based on the calibrated model and forward simulations using the Gillespie algorithm [19]. As ITS data are available immediately (i.e., without the week-long reporting lag of the CDC data), all 1 week forecasts are “nowcasts” [S1 Text: Calibration and Prediction].

### Scoring approach

We first produced 1 to 4 week model-based forecasts of the weighted ILI-related proportion of physician visits, (*ILI*_*prop*_), using the weighted ILI-related proportion of physician visits as reported by the CDC as a proxy in the model for new weekly cases; these data were assumed to have a 1 week reporting delay. We then compared these forecasts with predictions using as a proxy for new weekly cases the estimates of the weighted ILI-related proportion of physician visits, *ILI*_*prop*_, developed using ITS data, which do not have any reporting delay.

Using the scoring system of the CDC influenza prediction challenge [20, 21], we assessed the accuracy of the two sets of forecasts. This scoring system bins the proportion of the weighted ILI-related physician visits (*ILI*_*prop*_) in steps of 0.1 and sums the forecast posterior probability that falls into the bin containing the true value plus the five preceding and five following bins. We then compared the score of forecasts using CDC data that have a 1 week lag to the score of forecasts using estimates of the CDC data developed from real-time ITS influenza metrics. The gold-standard is CDC data that have a 1 week lag, but generally CDC data is published with a 1 to 2 week lag, and is often revised later as additional results become available.

## Results

We obtained a dataset of national influenza test results collected from a network of diagnostic machines named Virena from Quidel Corporation (ITS). ITS recorded over 805,000 test results between September 1st, 2015 and May 12, 2017. In the 2016-2017 influenza season, the ITS network included 3079 diagnostic machines spanning 1782 distinct locations in the United States.

**Fig 1:**
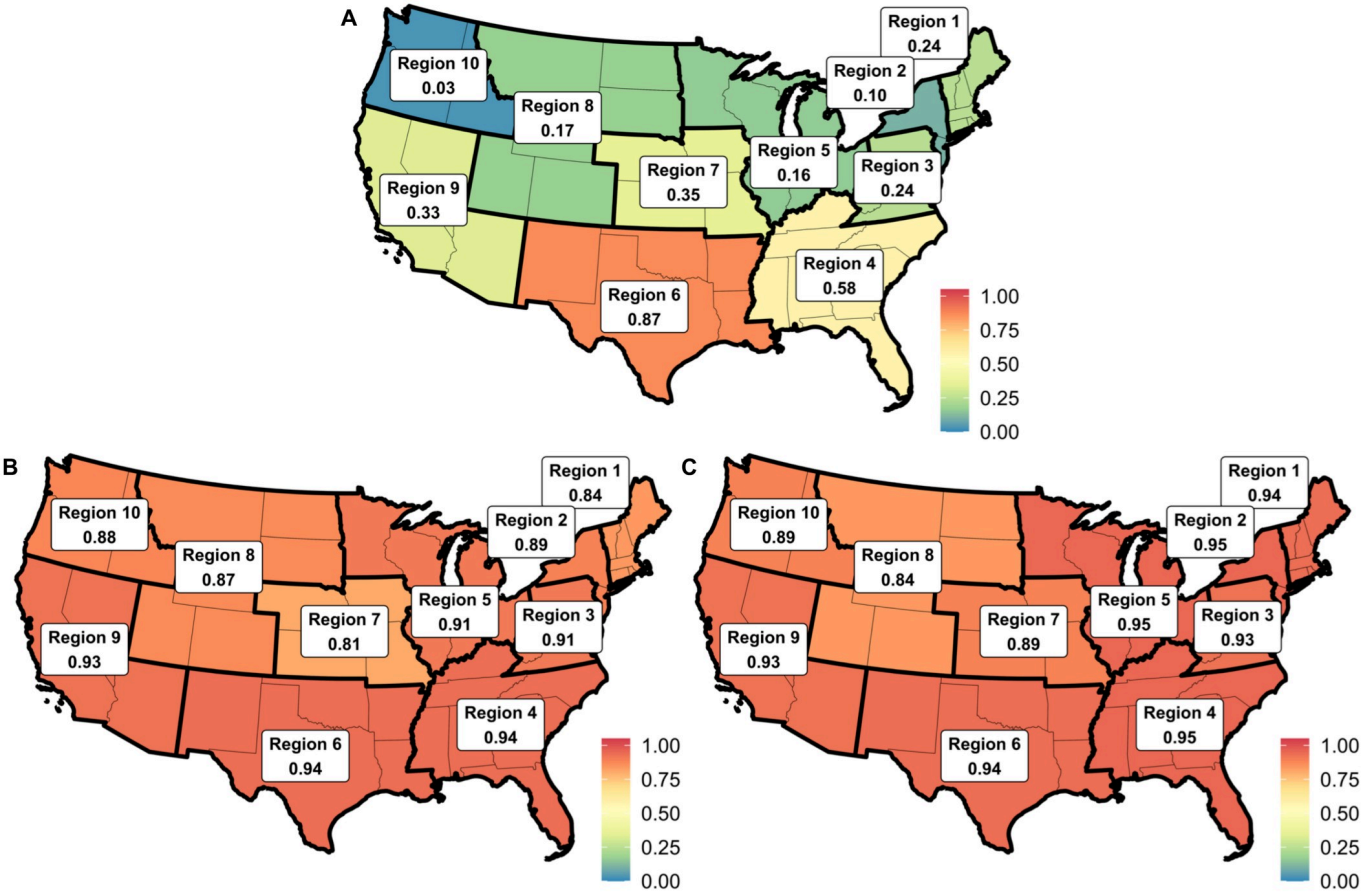
(A) Volume of the ITS network. Volume was determined by dividing the total number of ITS-reported influenza tests in a specified region per epi week by the total number of CDC-reported influenza tests in the same region per epi week and averaging this metric over the length of the analysis. (B) ITS Model 1 adjusted *R*^2^ values. Correlation between the estimated proportion of tests positive in the CDC surveillance system (WHO/NREVSS and ILINet) based on ITS Model 1 and the actual proportion of tests positive in the CDC surveillance system. (C) ITS Model 2 adjusted *R*^2^ values. Correlation between the estimated ILI-related proportion of physician visits in the CDC surveillance system (WHO/NREVSS and ILINet) based on ITS Model 2 and the actual ILI-related proportion of physician visits in the CDC surveillance system. These three continental U.S. maps show the volume of the ITS network, the adjusted *R*^2^ values for ITS Model 1, and the adjusted *R*^2^ values for ITS Model 2. While the volume of the ITS network varies per region, the adjusted *R*^2^ values for ITS Model 1 and ITS Model 2 are all high and above 0.80. Hawaii is in Region 9, and Alaska is in Region 10. The ITS network includes fewer influenza tests than the CDC surveillance system, and varies based on HHS region (a). The high correlation between the ITS Models’ estimates of the CDC metrics and the actual CDC metrics suggest that the ITS Models fit the true data well (b and c). Map obtained from [22].

The ITS dataset includes test results from 46 states. The data includes most areas in the CDC HHS Regions, with the exceptions of Maine, Rhode Island, Vermont, Idaho, Puerto Rico, U.S. Virgin Islands, and the District of Columbia. The ITS dataset is available in real-time and at relatively high geographic resolution. The national volume, or the average over the two influenza seasons of the total number of influenza tests as recorded by ITS for each epidemiological week divided by the total number of influenza tests as reported by the CDC for the corresponding epidemiological week, is 0.36, and the volume ranges from 0.87 in Region 6 (Arkansas, Louisiana, New Mexico, Oklahoma, and Texas) to 0.03 in Region 10 (Alaska, Idaho, Oregon, and Washington) (Fig 1a).

### Nowcasts

We first investigated the relationship between influenza metrics recorded by the ITS dataset and influenza metrics reported by the CDC for two measures of influenza activity: (1) the proportion of influenza diagnostic tests that are positive, and (2) a measure of influenza-related care seeking, the weighted ILI-related proportion of physician visits.

**Fig 2:**
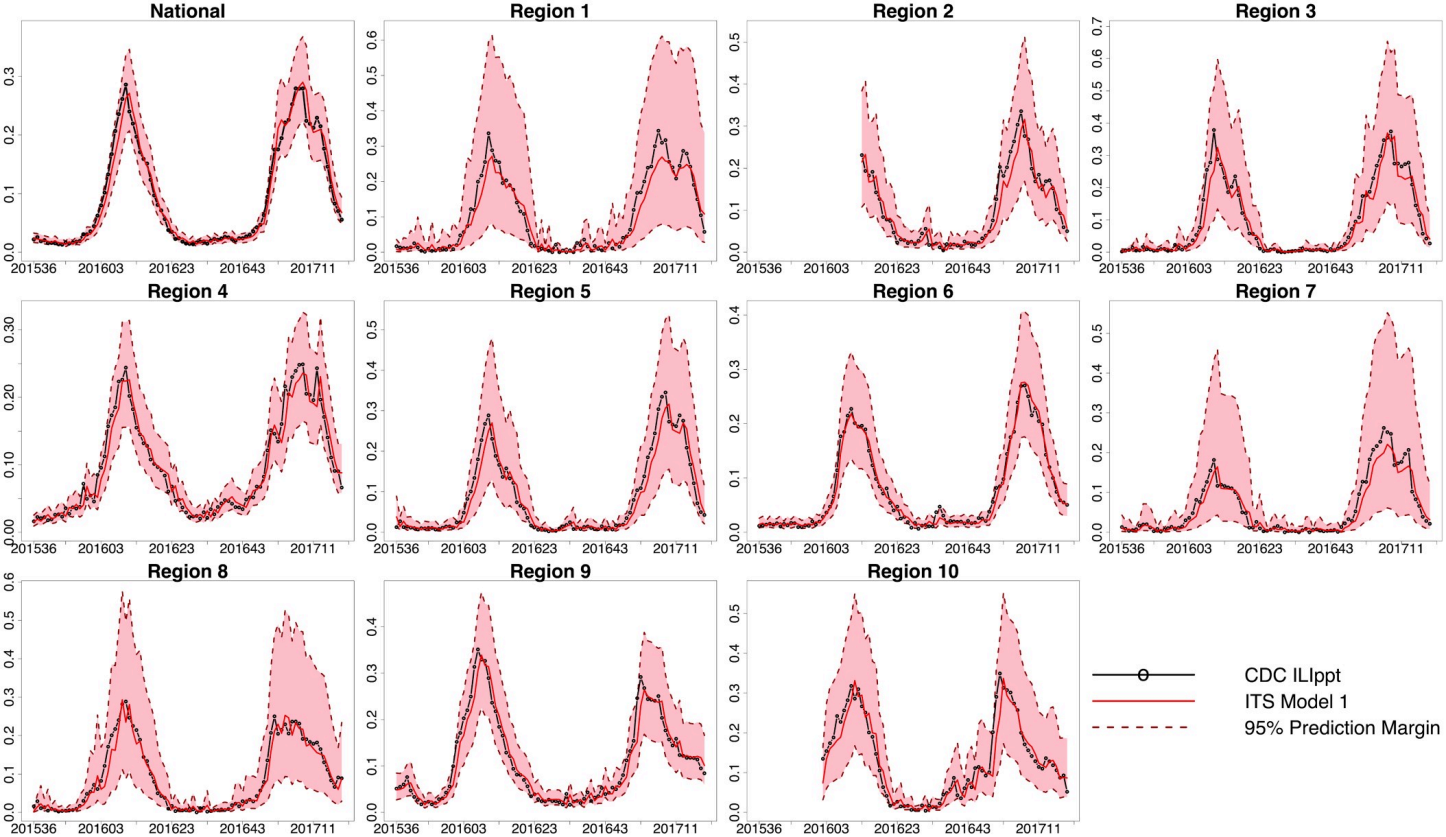
The estimated influenza tests that are positive developed from the ITS Model 1 track well the actual influenza tests that are positive as reported by the CDC. ITS Model 1 estimates the CDC proportion of influenza tests that are positive (*ILI*_*ppt*_(*t*_*a*_)) by using the proportion of influenza tests that are positive as recorded by ITS (*V*_*ppt*_(*t*_*a*_)), the CDC proportion of influenza tests that are positive with a 1-week lag (*ILI*_*ppt*_(*t*_(*a*−1)_)), and the absolute value of the difference between the proportion of tests that are positive as recorded by ITS with a 1-week lag and the proportion of influenza tests that are positive as reported by the CDC with a 1-week lag (|*V*_*ppt*_(*t*_(*a*−1)_) − *ILI*_*ppt*_(*t*_(*a*−1)_)|). Each graph shows two peaks with each peak relating to one flu season which occurs in the winter. The proportion of influenza tests that are positive is along the y-axis. The CDC proportion of influenza tests that are positive is in black, the ITS model estimates are in red, and the 95% prediction intervals are outlined by dark red dotted lines. The epidemiological week (epi week) is along the x-axis and spans from epi week 36 in 2015 to epi week 19 in 2017, except ITS data collection (and thus analysis) began later for Region 2 (epi week 13 in 2016 to epi week 19 in 2017) and Region 10 (epi week 2 in 2016 to epi week 19 in 2017). See Figure C in S1 Text for a visualization of raw data and estimates.

**Fig 3:**
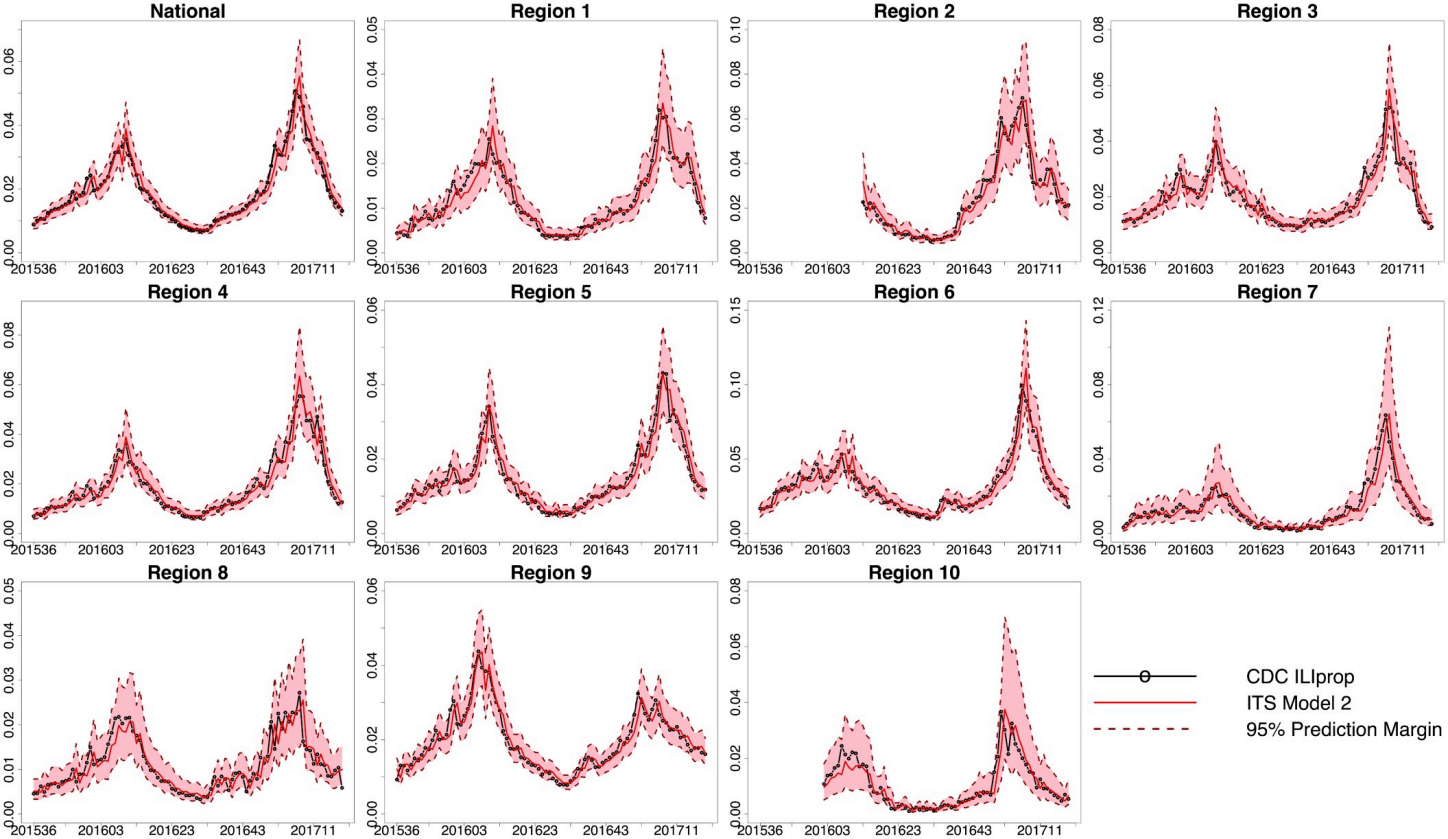
The estimated weighted ILI-related proportion of physician visits developed from the ITS Model 2 track well the actual weighted ILI-related proportion of physician visits as reported by the CDC. ITS Model 2 estimates the CDC weighted ILI-related proportion of physician visits (*ILI*_*prop*_(*t*_*a*_)) by using a metric developed from ITS data (the total number of influenza test results divided by the total number of test machines, *V*_*total*_(*t*_*a*_)), the weighted ILI-related proportion of physician visits as reported by the CDC with a 1-week lag (*ILI*_*prop*_(*t*_(*a*−1)_)), and the ITS metric (the total number of influenza test results divided by the total number of test machines) with a 1-week lag (*V*_*total*_(*t*_(*a*−1)_)). Each graph shows two peaks with each peak relating to one flu season. The weighted ILI-related proportion of physician visits is along the y-axis. The CDC weighted ILI-related proportion of physician visits are in black, the ITS model estimates are in red, and the 95% prediction intervals are outlined by dark red dotted lines. The epidemiological week (epi week) is along the x-axis and spans from epi week 36 in 2015 to epi week 19 in 2017, except ITS data collection (and thus analysis) began later for Region 2 (epi week 13 in 2016 to epi week 19 in 2017) and Region 10 (epi week 2 in 2016 to epi week 19 in 2017). See Figure D in S1 Text for a visualization of raw data and estimates.

For all geographic regions, the estimates developed using ITS data tracked the influenza metrics as reported by the CDC (Figs 2 and 3). We found a strong positive correlation between the proportion of influenza tests that are positive as reported by the CDC (*ILI*_*ppt*_) and the proportion of influenza tests that are positive as recorded by ITS (*V*_*ppt*_) on both national and regional levels, with all relationships demonstrating an adjusted *R*^2^ value greater than 0.80 (Fig 1b). Similarly, we found a strong positive correlation between the proportion of all physician visits that are ILI-related as reported by the CDC (*ILI*_*prop*_) and a metric developed by dividing the total number of tests by the total number of machines as recorded by ITS (*V*_*total*_) on both national and sub-national levels (all adjusted *R*^2^ values were greater than 0.80) (Fig 1c).

Previous publications compared their models to a baseline model that uses only historical CDC influenza data [3]. Thus, we developed a baseline model using only historical CDC data, using the gold standard of a 1 week lag in CDC data, to estimate current CDC metrics. For most regions, the ITS model estimated the current CDC influenza metric statistically better than a respective baseline model for both CDC metrics estimated (i.e., the proportion of influenza tests that are positive and the weighted ILI-related proportion of physician visits) [S1 Text: ANOVA].

### Forecasts

After determining that ITS data can accurately estimate current CDC influenza metrics, we then investigated whether the earlier availability of such estimates can improve the performance of model-based projections of epidemic behavior. We used estimates of current weighted ILI-related proportion of physician visits developed by using ITS data (*ILI*_*prop*_) to predict the actual CDC weighted ILI-related proportion of physician visits 1, 2, 3, and 4 weeks in the future. Figure B in S1 Text shows retrospective forecasts for the 2016/17 and 2017/18 season. To avoid contamination with unavailable data, the ITS nowcasting model for the 2016/17 season is only trained with 2015/16 season’s data and the ITS nowcasting model for the 2017/18 season is only trained with 2015/16 and 2016/17 season’s data. We omit the first season for Regions 2 and 10 as their data recording started later.

### Scoring

We evaluated the performance of the humidity-based SIRS models in making projections 1, 2, 3, and 4 weeks in the future. We found that estimates of the weighted ILI-related proportion of physician visits developed using ITS data (*ILI*_*prop*_) improve predictions compared to using historical CDC weighted ILI-related proportion of physician visits that have a 1 week lag. Using estimates of this metric (developed by using ITS data) in the humidity-based SIRS model improved the 1 week prediction accuracy by 30% lower log-score in average over regions and seasons compared to predictions developed from a model only using CDC data with a one-week lag. When forecasting 2 weeks into the future, using the estimated metric improves the prediction accuracy by 31% compared to using CDC data with a one-week lag. Similarly, for 3 weeks, the prediction accuracy improves by 17% and for 4 weeks, improves by 10% (Figure B in S1 Text).

For 76 out of 80 combinations of season, year, region, and forecast horizon, using estimates of the CDC metric developed by using ITS data improves prediction accuracy compared to using the CDC metric with a 1 week lag. As the prediction horizon increases, the prediction accuracy decreases, and this improvement using estimates instead of historical CDC data is less substantial, as the relative gain of having data available 1 week earlier decreases with longer forecasting intervals.

### Benchmarking to other real-time data sources

**Fig 4:**
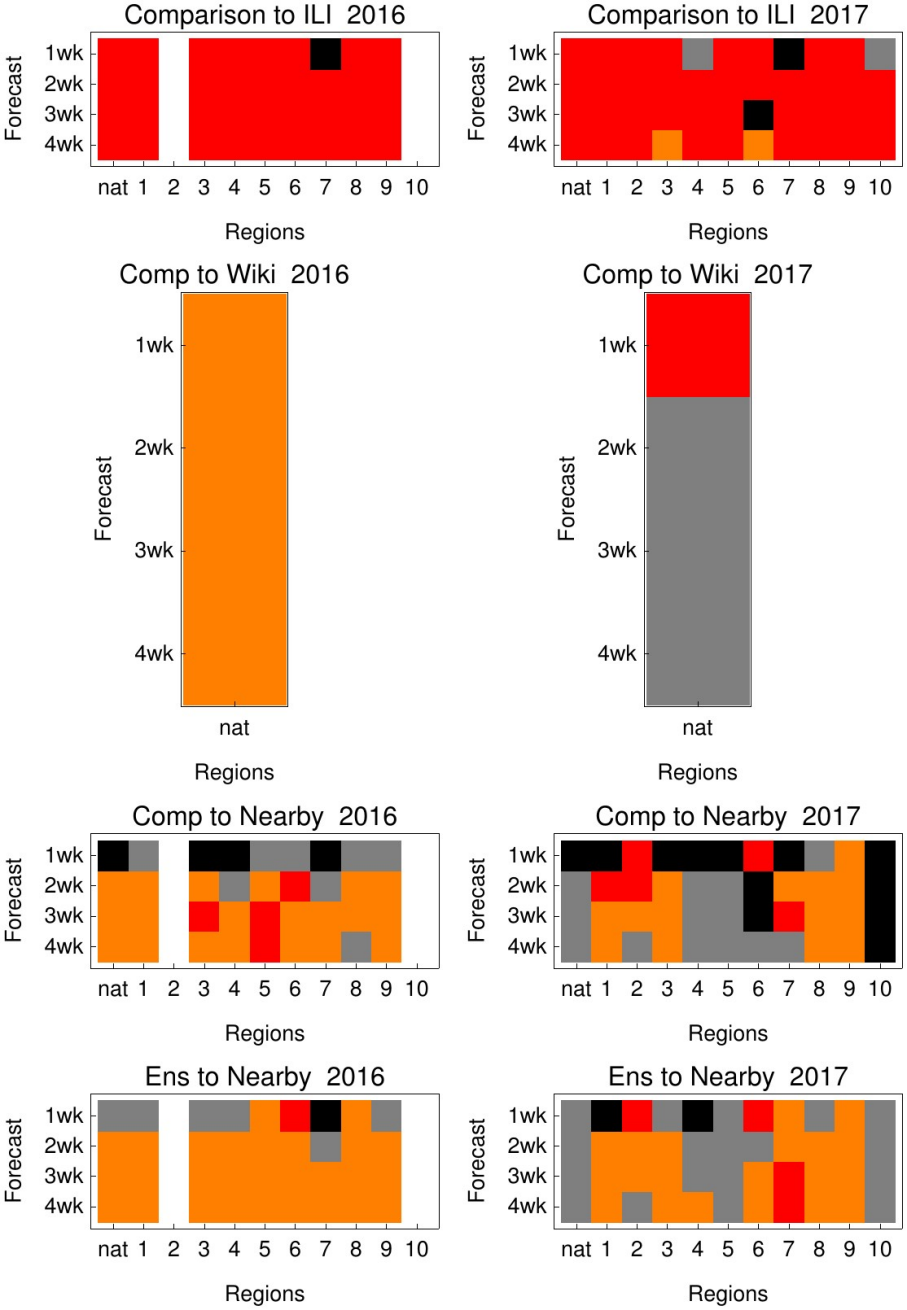
Comparing ITS data based forecast to other real-time data sources. Each panel compares the log-score difference of ITS forecasts to forecasts based on another data source. Red color: > 0.1 average log-score improvement associated with ITS; Orange: (0, 0.1]; Grey: [−0.1, 0); Black: < −0.1 worse. Values of zero did not occur and white color stands for Reg 2 and 10 in 2016 when no forecasts where calculated. First row: we see clear improvement of ITS compared with ILI as in the Fig 5. Second row: ITS compared with nowcasting source Wikipedia. Third row: ITS compared with ILINearby. Third row: Comparing an ensemble of ITS + ILINearby with Nearby. These comparisons demonstrate that ITS is a valuable addition to presently available nowcasting data sources.

We then compared the performance of forecasts based on ITS data to forecasts based on other data sources with timelier availability. First, we compared against Wikipedia data [23] which has successfully been used for forecasting in several previous publications [5, 24]. Fig 4 (second row) shows that ITS data improves the majority of forecasting target—region combinations. However, Wikipedia data is only available on a national level.

Therefore, we chose a second benchmark that is also available on a regional level and is a very challenging benchmark; ILINearby [25, 26] is a nowcasting system based on multiple real-time data sources including Google, Twitter and Wikipedia. We do not expect to outperform this nowcasting system on all targets, however, we see that ITS performs better on some target-region combinations. This demonstrates the usefulness of including ITS data as an additional data source in current nowcasting efforts.

As ensemble models have shown strong performance in influenza forecasting [27, 28], this also leads to the question: how much can be gained by combining ILINearby data and ITS data within an ensemble model. As only few seasons of ITS are currently available, we were restricted to using naive ensembles (average between both models). Nevertheless, the naive ensemble model appears to have superior performance in the majority of forecasts.

A 1 week reporting delay for the CDC’s ILI is an approximation and, therefore, we performed an additional analysis for the 2016/2017 season using the exact reporting days of CDC’s ILI. In this additional analysis we also find that the new ITS data source improves forecasts (see Figure A in S1 Text).

## Discussion

A distributed network of diagnostic machines operated by Quidel Corporation automatically reports de-identified influenza test results which are then compiled in the ITS dataset [8]. We evaluated the ability of ITS, a direct signal of influenza activity, to estimate and predict influenza metrics on national and sub-national levels in the United States.

**Fig 5:**
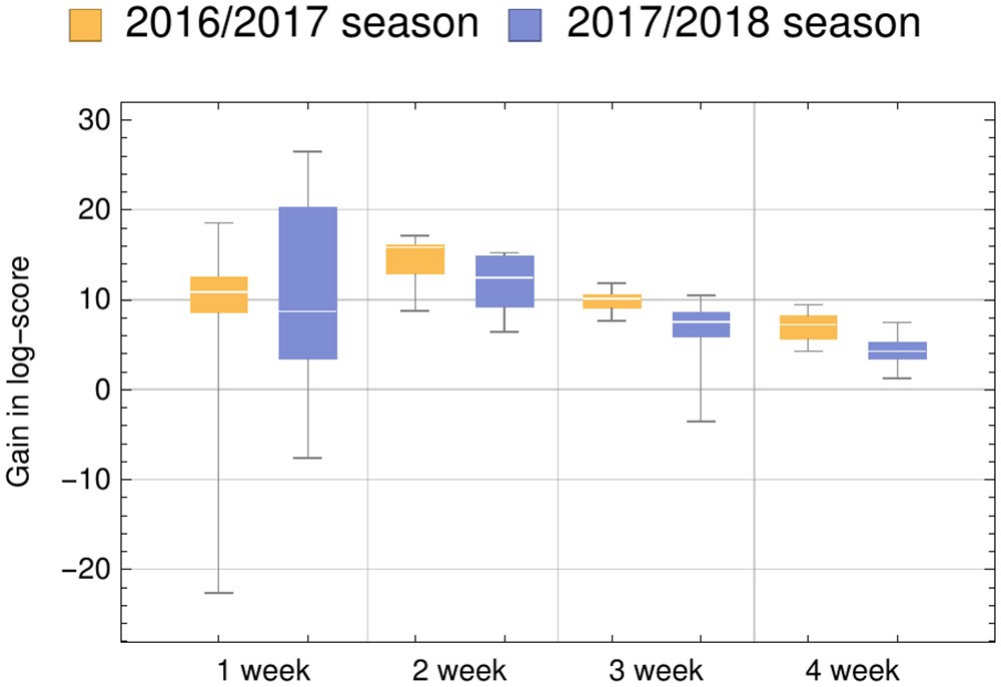
ITS data can be used to improve the forecasting accuracy of current influenza trends. Total seasonal log-score gain for predictions using real-time estimates using the ITS data compared to CDC data with a 1 week lag for different forecasting horizons for the 2016-2017 and 2017-2018 season for one region. The model shows the most improvement (i.e., gain in log-score) compared to the CDC data with a 1 week lag when predicting 1 week into the future, but even in predictions with larger horizons, predictions using ITS data were better than predictions using CDC data with a 1 week lag. Boxplots summarizes results from the 11 different geographical areas. Figure B in S1 Text shows details for all areas.

We developed linear logistic models using ITS data and historical CDC data to: (1) estimate the proportion of influenza tests that are positive as reported by the CDC by using proportion of influenza tests that are positive as recorded by ITS (Fig 2); and (2) estimate the weighted ILI-related proportion of all physician visits as reported by the CDC by using a metric developed from ITS data (the total number of influenza test results divided by the total number of test machines as recorded by ITS) (Fig 3). We then used the estimated weighted ILI-related proportion of all physician visits to accurately predict the actual CDC reported weighted ILI-related proportion of physician visits 1, 2, 3, and 4 weeks in the future (Fig 5).

In previous research using indirect influenza signals such as Google Flu Trends [4, 6, 29], Wikipedia [5, 23], and Twitter [3, 7], researchers first established that the indirect influenza signal could track the CDC influenza metric. We have demonstrated that influenza test results can track well the CDC metrics as shown in Fig 2 and in Fig 3. In addition, statistical tests showed that the addition of the ITS data in the model statistically improved the model fit to the actual CDC data in most regions [S1 Text: ANOVA]. Further, we found that using real-time data on influenza test results allowed us to develop better model predictions of influenza activity than models that used only historical CDC data (Figure B in S1 Text).

In addition, ITS data also allows us to improve predictions compared to other indirect real-time data sources such as Wikipedia [23] or ILINearby [25] as Fig 4 shows, hereby indicating that use of ITS can contribute to the accuracy of nowcasting efforts.

Use of ITS data allowed us to accurately estimate and predicted CDC influenza metrics and to avoid some limitations associated with previous studies using indirect signals of influenza activity. These indirect signals are subject to changing behaviors of the public that are not directly caused by influenza activity, whereas ITS and the CDC influenza surveillance system use direct indicators of influenza activity. The Google Flu Trends model overestimated influenza epidemics possibly because the models included irrelevant search terms [6], a potential flaw we avoided by use of a more direct signal of influenza activity. Models using Wikipedia face issues of non-independence as articles may be correlated [5]. With few possible exceptions, ITS test results are independent. In addition, the Wikipedia model is not specific to the United States as article views on influenza activity may originate from other English-speaking countries [23]. The ITS network compiles data transmitted from machines located only in the United States [8]. In addition, Wikipedia and Twitter face drawbacks in geographic resolution as these data sources are accessible only nationally or in limited areas [5, 7], while the ITS dataset includes location, and thus, we were able to develop models on multiple geographic levels: at the national level and in the 10 HHS Regions.

Future work could also compare Electronic Health Record data based forecasts (such as in [30]) to the performance of ITS data based forecasts identifying similarities and differences and, with that, building ensemble models exploiting the strength of both sources.

We were able to accurately estimate and predict influenza trends in the United States using ITS data. However, some physicians may suggest testing for influenza at higher rates than others, thus possibly affecting the correlation between influenza tests results and ILI activity. The density of the test machines may also not be directly proportional to the density of the population. In addition, test results are not necessarily independent of each other as the same patient may have several tests performed per ILI; we assumed any non-independence was negligible. In addition, the data collection began later for Region 2 and Region 10, restricting the time span of our analysis for those two regions. Finally, while ITS data is not subject to changing behavior by the general public, the data is obtained from commercial machines and therefore is subject to changing behaviors relating to the buying and selling of these machines. A potentially economically-driven change in behavior could include targeting the sale of these machines to physicians who are more likely to test for influenza in order to increase profits on testing supplies.

Our results showing the improvement of forecasts by using ITS data were obtained with a SIRS humidity-based model [15] and the MSS calibration and prediction framework [16]. We would encourage further research in order to investigate the effects of incorporating ITS data in other influenza forecasting frameworks.

The ITS dataset included most areas defined by the CDC HHS Regions, had 36% as many test results as the CDC’s surveillance network, and began in September 2015, limiting our analysis to two complete influenza seasons. In addition, we are unable to determine conclusively if there was overlap in terms of actual clinics and patients. Moreover, our analysis showed that regions with lower volume did not necessarily experience lower model performance (as measured by adjusted *R*^2^). As the ITS network continues to expand both in scope (i.e., volume), spatial resolution, and time, some of these constraints may be addressed.

We note a stronger performance of ITS for the 2016/17 season than for the 2017/18 season compared to the benchmarks. As influenza is different in every season, forecasts will also be different in absolute log-score as well as relative log-score for varying methods and data sources. Once, sufficient season are available, statistical tests can be applied to deepen the comparison between approaches.

In subsequent studies, we plan to use ITS data to develop real-time estimates in strain-specific or age-specific model forecasting. Secondary strains have been hypothesized to be responsible for heightened levels of influenza activity late in the influenza season [5]. Thus, these estimates could be used in models that incorporate multiple strains of influenza to better predict influenza burden. As influenza disproportionately affects young children and the elderly [7], an age-stratified model may also improve model forecasts. Furthermore, recent work [31] suggest that exploiting cross-regional correlation increases forecasting accuracy. The high geographical resolution of ITS data makes it a well-suited candidate to apply this method in the future.

We used ITS data to develop accurate and timely estimates and predictions of influenza activity, which are essential for public health planning and mitigating influenza burden.

## Supporting information

S1 Text S1 Text provides supporting information. (PDF)
