# Supplementary material for: Tracking and predicting U.S. influenza activity with a real-time surveillance network
Source: PLoS Comput Biol. 2020 Nov 2;16(11):e1008180. doi: 10.1371/journal.pcbi.1008180 (PMC7707518; doi:10.1371/journal.pcbi.1008180)
Supplement: S1 Text — (PDF) [file pcbi.1008180.s001.pdf]

Supporting Information (SI) – S1 Text – for Tracking and predicting U.S. influenza activity with a real-time surveillance network

Sequoia I. Leuba[1], Reza Yaesoubi[2], Marina Antillon[3,4], Ted Cohen[1], Christoph Zimmer[1*]

1 Epidemiology of Microbial Diseases, Yale School of Public Health, New Haven, CT, USA
2 Health Policy and Management, Yale School of Public Health, New Haven, CT, USA
3 Household Economics and Health Systems Research Unit, Swiss Tropical and Public Health Institute, Basel, Switzerland
4 University of Basel, Basel, Switzerland

Cleaning ITS

Quidel Corporation sent the ITS dataset partially on a weekly basis and partially in bulk to our research group, and the datasets were merged. Invalid test results were removed from the dataset. The first 4 weeks available from Quidel Corporation for Region 2 were not included in the analysis because of missing data for the number of positive test results, the overall number of test results, and the number of test machines. Thus, the data analysis for Region 2 began at the 13th epidemiological week of 2016 instead of the 9th epidemiological week of 2016. Data for the number of positive test results, the overall number of test results, and the number of test machines were missing for fewer than five weeks in 2016 for Region 10. We imputed values for missing tests and machines in those weeks using the average of the epidemiological week before and after. For example, for the number of positive test results in the 7th epidemiological week in 2016, we used the rounded mean of the number of positive test results in the 6th epidemiological week in 2016 and the number of positive test results in the 8th epidemiological week in 2016.

ANOVA

We developed a baseline model similar to a previous publication [3] which used historical CDC influenza data to estimate current CDC metrics. Following the gold standard procedure, we estimated the current CDC week's metric using only the previous week's CDC metric. As this baseline model was nested in the ITS model, we were able to compare the two using ANOVA in order to determine if including ITS data improved upon the baseline model. The ANOVA tests whether the addition of the ITS term leads to a significant improvement over the null model. The null hypothesis is that the null model estimates the current CDC week's metric as well as the ITS model. The alternative hypothesis is that the null model estimates the current CDC week's metric differently than the ITS model. A low P-value indicates that the ITS model estimates the current CDC week's metric better than the null model. We used an α of 0.05 as the threshold for statistical significance. For most of our models, the P-value for the ANOVA was less than 0.05 (Table A in S1 Text).

The baseline model for the proportion of diagnostic tests was the relationship between the historical proportion of diagnostic tests that are positive as recorded by CDC ($ILI_{ppt}(t_{(a-1)})$) and the current proportion of tests that are positive as reported by the CDC at the present ($ILI_{ppt}(t_a)$) (Equation 3)

$$logit(ILI_{ppt}(t_a)) = \beta_1 logit(ILI_{ppt}(t_{(a-1)})) + \epsilon \quad (3)$$

where β_1 is the coefficient; t is the time variable with the current epidemiological week as t_a and the previous epidemiological week as $t_{(a-1)}$; and ϵ is the error term.

The baseline model for the weighted ILI-related proportion of physician visits was the relationship between the historical weighted proportion of all physician visits that are ILI-related as reported by the CDC ($ILI_{prop}(t_{(a-1)})$) and the current weighted proportion of all physician visits that are ILI-related as reported by the CDC ($ILI_{prop}(t_a)$) (Equation 4)

$$logit(ILI_{prop}(t_a)) = \beta_1 logit(ILI_{prop}(t_{(a-1)})) + \epsilon \quad (4)$$

where β_1 is the coefficient; t is the time variable with the current epidemiological week as t_a and the previous epidemiological week as $t_{(a-1)}$; and ϵ is the error term.

Table A in S1 Text. ANOVA P-values comparing ITS to Baseline Models

**Region**	**Model 1**	**Model 2**
National	< 0.001	< 0.001
Region 1	< 0.001	0.004
Region 2	0.131	0.020
Region 3	0.316	< 0.001
Region 4	0.004	< 0.001
Region 5	0.955	< 0.001
Region 6	< 0.001	< 0.001
Region 7	0.008	0.176
Region 8	< 0.001	0.137
Region 9	0.128	0.014
Region 10	0.164	0.058

Computational model

We used a humidity-based susceptible-infected-recovered-susceptible (SIRS) influenza model published by Shaman et al., 2013 [32]:

$$S \xrightarrow{\beta(t)*S*I/N} I \quad (5)$$

$$I \xrightarrow{I/\gamma} R \quad (6)$$

$$R \xrightarrow{R/\alpha} S \quad (7)$$

with an average duration of immunity α, a mean infectious period γ and a transmission rate $\beta(t)$. The transmission rate $\beta(t)$ is defined as $\beta(t) = R_0(t)/\gamma$ with an $R_0(t) = \exp(-180q(t)+\log(R_{0max}-R_{0min}))+R_{0min}$ where R_{0max} and R_{0min} describe the maximal and minimal daily reproductive number. The function q is used to describing the absolute humidity. In [17], we independently reconstructed the specific humidity dataset following the method detailed by the Text S1 of [15]. As in [16] the specific humidity dataset was compiled from the primary forcing dataset from Phase 2 of the North American Land Data Assimilation System (NLDAS-2) [33]. This data was obtained though the National Center for Environmental Prediction North American Regional Reanalysis. The hourly data is available on a 0.125°grid from 1979 to present. After extracting the specific humidity data for 121 cities, we then averaged the hourly data to obtain a daily climatology for each city from 1979 to 2016. We then averaged data for each year from 1979 until 2016 to develop an average year-long humidity profile for each city. To develop regional or national humidity profiles, we took the average of year-long humidity profiles of applicable cities.

For a proxy for the weekly number of incident infections, we used the CDC ILI data with a one-week lag or the real-time estimate of the CDC ILI data using ITS data, and it is linked to the number of people in the computational model that transition from susceptible to infectious in each time interval. Like previous analyses [5], we do not account for different influenza strains or non-influenza causes of ILI.

Calibration and Prediction

We summarized our existing knowledge in a prior distribution $\pi_0(\theta)$ over the parameter θ. Whenever new observations y_i at time points t_i occurs, we iteratively updated our knowledge by multiplying the prior with

the probability to observe y_i:

$$\pi_i(\theta|y_i, y_{i-1}, \dots, y_1) \;=\; \pi_{i-1}(\theta|y_{i-1}, \dots, y_1)\mathcal{P}(y_i|y_1, \dots, y_{i-1}; \theta) \tag{8}$$

*[Posterior at time t_i] = [Prior at time t_i] * [probability to observe y_i].* The previous posterior serves as the prior for the next observation. As there are no observations for the initial time t_0, we set $\pi_0(\theta|y_0) = \pi_0(\theta)$.

We calculated the probability $\mathcal{P}$ in equation (8) by conditioning at the epidemic state ν_i at time $t - i$ and ν_{i-1} at time t_{i-1}:

$$\mathcal{P}(y_i|y_1, y_2, \dots, y_{i-1}; \theta) \;= \sum_{\nu_i \in \Omega_i} \sum_{\nu_{i-1} \in \Omega_{i-1}} P(y_i|\nu_i, \nu_{i-1}; \theta) p(\nu_i|\nu_{i-1}; \theta) \Pi(\nu_{i-1}|y_1, y_2, \dots, y_{i-1}; \theta). \tag{9}$$

*[Probability for current observation conditioned on history] = [Sum over all possible current states] [Sum over all possible previous states] [Probability for observation conditioned on specific current state] * [Transition probability to move from specific previous to specific current state] * [Belief state probability to be in specific previous state given history]* $\Pi(\cdot|y_1, y_2, \dots, y_i)$ denotes the "belief state" which describes the probability distribution over the epidemic state ν_{i-1}. Ω_i is the support of the belief state at time t_i and p is the transition probability to move from state ν_{i-1} at time t_{i-1} to state ν_i at time t_i. The observation probability P maps the state ν_i to the observation y_i accounting for additional uncertainty in the data such as reporting errors.

We used a linear noise approximation (LNA) to approximate the transition probability p [17,18,34]. The LNA approximates the distribution of $\nu_i|\nu_{i-1}$ with a normal distribution of which the mean is the solution of the ordinary differential equation representation of the system on the interval $[t_{i-1}, t_i]$ and the covariance is the solution of the LNA system (equation 5 and 6 of [17]).

In order to calculate initial values for the ODE and LNA system at the beginning of each interval, we employed a state estimation procedure as outlined in the MSSa version of [16]. This state estimation procedure incorporates the information provided by each new observation, and uses the state as a state estimates that maximizes the path probability from a previous state estimate to the current observation. All prediction was performed using Mathematica version 10.4 [10].

Predictions are based on forward simulations using a Gillespie algorithm [19] in the software COPASI [35]. The forward simulations are initialized by samples from the posterior parameter distribution π and the posterior state distribution Π as in [16].

Benchmark results taking into account exact time stamps of reporting

CDC's ILI is reported with a one week delay and, then, is still corrected for several more weeks before it stabilizes to its final value. Therefore, we repeated the analysis carefully considering a) the time stamps of reporting of ILI for when calculating ITS nowcasts, and b) the exact time stamps of ITS data being delivered.

We used the time period from epiweek 40 in 2015 to epiweek 19 in 2016 for training the regression model. As this is a very short time, we note that training region-specific models is not feasible. Therefore, we trained one model averaging across all regions being aware that this will result in a loss in accuracy in general, but otherwise would not be possible due to the limited amount of data.

Most of the ITS data which was weekly was sent to us on Friday. We selected Monday as the due date of forecasts as the CDC's ILI forecasting challenge also uses this due date (except for the post-Christmas and post-New Year week, when the CDC delays this due date and we did, therefore, correspondingly). Whenever, ITS data was not available for the full corresponding epi week until the due date, we used the available days and up-weighted them (e.g. for 5 available days, multiplied by 7/5.

Nonetheless, we see a valuable contribution of the ITS data source in Figure A in S1 Text for the test time starting in epi week 46 in 2016 and leading to epi week 19 in 2017.

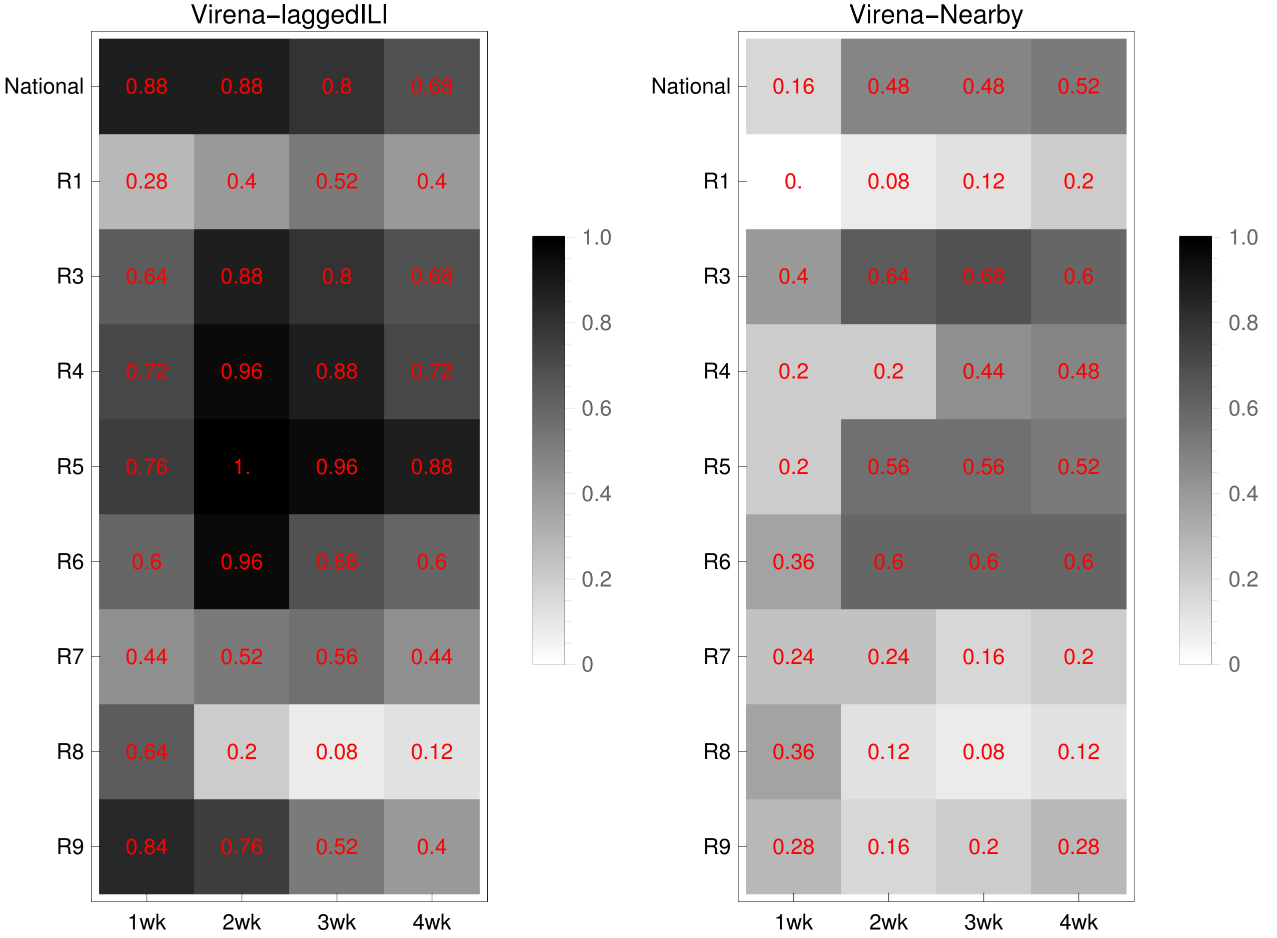

Fig A in S1 Text. Benchmark results considering exact time stamps of reporting: Each panel shows comparison between two data scenarios, columns are geographical regions, and rows are forecasting week targets. Red number is the fraction of first data scenario results better than the second in terms of log-score in the test time from epi week 46 in 2016 to epi week 19 in 2017.

ITS improves clearly over lagged ILI. ILINearby is on average better than ITS but not in all cases. For some target-region combinations, ITS is better than more than half the forecasts, for others, it performs worse but rarely more than 10% worse. As ILINearby is an extremely challenging test case, this shows that ITS is a promising addition to the currently available data sources.

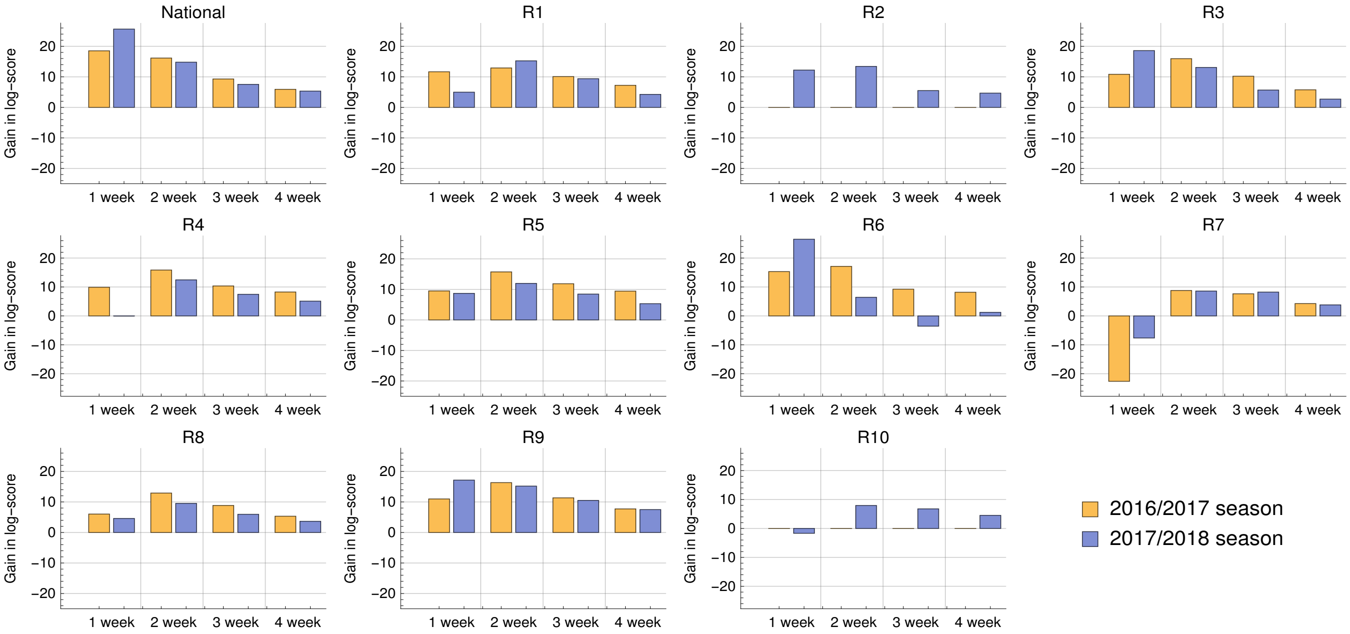

Fig B in S1 Text. ITS data can be used to improve the forecasting accuracy of current influenza trends. Each panel shows the total seasonal log-score gain for predictions using real-time estimates using the ITS data compared to CDC data with a 1 week lag for different forecasting horizons for the 2016-2017 and 2017-2018 season for one region.

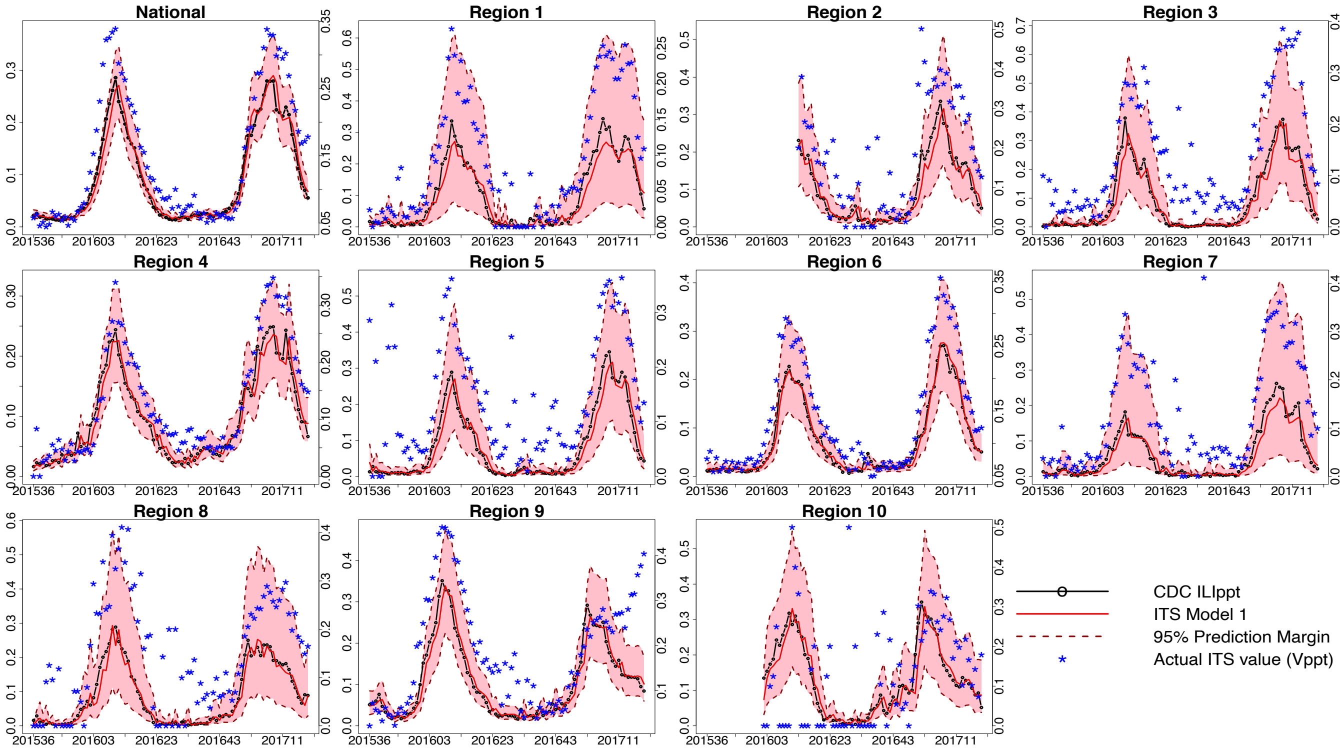

Fig C in S1 Text. The proportion of influenza tests that are positive as recorded by ITS ($V_{ppt}(t_a)$) track moderately well with both the estimated influenza tests that are positive developed from the ITS Model 1 and the actual influenza tests that are positive as reported by the CDC. The real-time ITS metric value ($V_{ppt}(t_a)$) is shown overlaid on ITS Model 1 estimates of the CDC proportion of influenza tests that are positive and the actual CDC proportion of influenza tests that are positive. The epidemiological week (epi week) is along the x-axis and spans from epi week 36 in 2015 to epi week 19 in 2017, except ITS data collection (and thus analysis) began later for Region 2 (epi week 13 in 2016 to epi week 19 in 2017) and Region 10 (epi week 2 in 2016 to epi week 19 in 2017). The proportion of influenza tests that are positive is along the left y-axis, and the real-time proportion of influenza tests that are positive as recorded by ITS ($V_{ppt}(t_a)$) is along the right y-axis. ITS Model 1 estimates the CDC proportion of influenza tests that are positive ($ILI_{ppt}(t_a)$) by using the proportion of influenza tests that are positive as recorded by ITS ($V_{ppt}(t_a)$), the CDC proportion of influenza tests that are positive with a 1-week lag ($ILI_{ppt}(t_{(a-1)})$), and the absolute value of the difference between the proportion of tests that are positive as recorded by ITS with a 1-week lag and the proportion of influenza tests that are positive as reported by the CDC with a 1-week lag ($|V_{ppt}(t_{(a-1)}) - ILI_{ppt}(t_{(a-1)})|$). The CDC proportion of influenza tests that are positive are in black, the ITS model estimates are in red, the 95% prediction intervals are outlined by dark red dotted lines, and the real-time proportion of influenza tests that are positive as recorded by ITS ($V_{ppt}(t_a)$) are the blue stars.

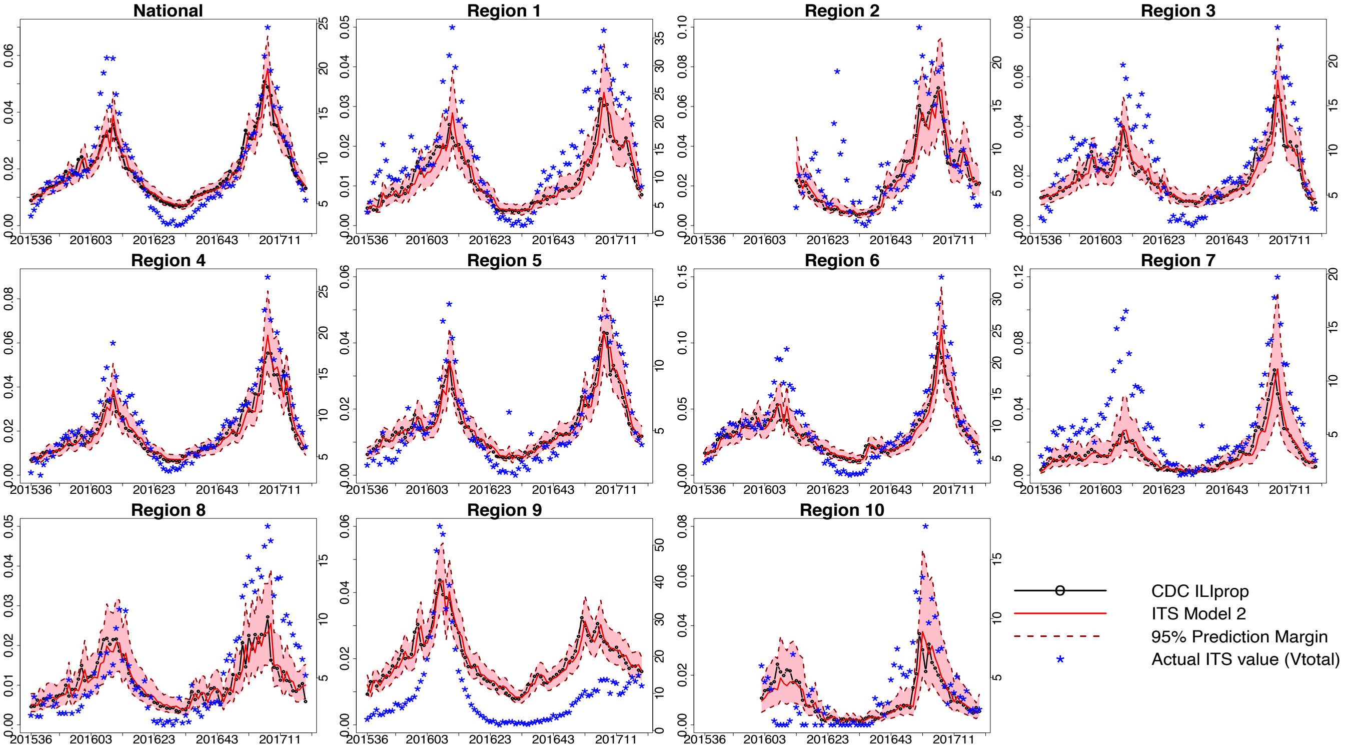

Fig D in S1 Text. The ITS metric value ($V_{total}(t_a)$, the total number of influenza test results divided by the number of influenza test machines as recorded by ITS) tracks moderately well both the estimated weighted ILI-related proportion of physician visits developed from the ITS Model 2 and the actual weighted ILI-related proportion of physician visits as reported by the CDC. The real-time ITS metric value ($V_{total}(t_a)$) is shown overlaid on ITS Model 2 estimates of the weighted ILI-related proportion of physician visits and the actual CDC weighted ILI-related proportion of physician visits. The epidemiological week (epi week) is along the x-axis and spans from epi week 36 in 2015 to epi week 19 in 2017, except ITS data collection (and thus analysis) began later for Region 2 (epi week 13 in 2016 to epi week 19 in 2017) and Region 10 (epi week 2 in 2016 to epi week 19 in 2017). The weighted ILI-related proportion of physician visits is along the left y-axis, and the real-time ITS metric value ($V_{total}(t_a)$, the total number of influenza test results divided by the total number of test machines) is along the right y-axis. The ITS Model 2 estimates the CDC weighted ILI-related proportion of physician visits ($ILI_{prop}(t_a)$) by using a real-time metric developed from ITS data ($V_{total}(t_a)$, the total number of influenza test results divided by the total number of test machines), the weighted ILI-related proportion of physician visits as reported by the CDC with a 1 week lag ($ILI_{prop}(t_{(a-1)})$), and the ITS metric with a 1 week lag ($V_{total}(t_{(a-1)})$). The CDC weighted ILI-related proportion of physician visits are in black, the ITS model estimates are in red, the 95% prediction intervals are outlined by dark red dotted lines, and the real-time ITS metric value ($V_{total}(t_a)$) are the blue stars.

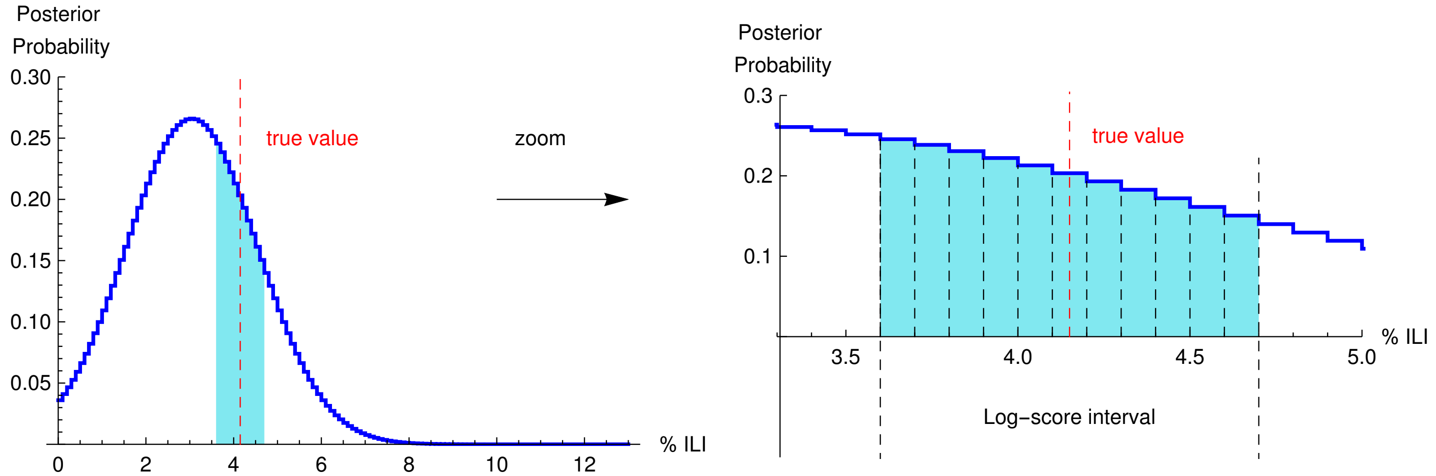

Fig E in S1 Text. Illustration of the log-score. Left side full picture, right side zoom in of the prediction posterior distribution. x-axis % ILI, y-axis posterior probability. Posterior distribution binned in steps of 0.1% ILI. The CDC's influenza forecasting challenge [20] uses a log-score that sums the probability of the bin containing the true value plus the five preceding and five following bins. The log-score is then calculated as the natural logarithm of this probability.
